# Systematic analysis of molecular mechanisms for HCC metastasis via text mining approach

**DOI:** 10.18632/oncotarget.14692

**Published:** 2017-01-17

**Authors:** Cheng Zhen, Caizhong Zhu, Haoyang Chen, Yiru Xiong, Junyuan Tan, Dong Chen, Jin Li

**Affiliations:** ^1^ Beijing 302 Hospital, Beijing, 100039, China

**Keywords:** hepatocellular carcinoma, metastasis, text mining

## Abstract

**Objective:**

To systematically explore the molecular mechanism for hepatocellular carcinoma (HCC) metastasis and identify regulatory genes with text mining methods.

**Results:**

Genes with highest frequencies and significant pathways related to HCC metastasis were listed. A handful of proteins such as EGFR, MDM2, TP53 and APP, were identified as hub nodes in PPI (protein-protein interaction) network. Compared with unique genes for HBV-HCCs, genes particular to HCV-HCCs were less, but may participate in more extensive signaling processes. *VEGFA*, *PI3KCA*, *MAPK1*, *MMP9* and other genes may play important roles in multiple phenotypes of metastasis.

**Materials and methods:**

Genes in abstracts of HCC-metastasis literatures were identified. Word frequency analysis, KEGG pathway and PPI network analysis were performed. Then co-occurrence analysis between genes and metastasis-related phenotypes were carried out.

**Conclusions:**

Text mining is effective for revealing potential regulators or pathways, but the purpose of it should be specific, and the combination of various methods will be more useful.

## INTRODUCTION

Hepatocellular carcinoma (HCC) is the second cause of death from malignancy tumor and the sixth most prevalent cancer worldwide [1]. Like many other types of malignance tumor, metastasis was considered the primary cause for treatment failure and HCC-associated mortalities [2, 3]. Understanding the molecular mechanisms involved in HCC metastasis would facilitate to develop novel strategies, such as personalized therapies and molecular-targeted drugs, which may help to improve the rate of survival.

A large number of genes and proteins have been examined for HCC metastasis. However, since metastasis is thought to be a multi-step process regulated by sophisticated molecular network, it is necessary to systematically assess the significance of each gene and find out the most important regulators.

Nowadays, text mining (TM) technology is increasingly applied for data analysis. In this study, we employed text mining technology and other bioinformatics methods to perform systematic analysis toward published articles, in order to figure out the critical genes and pathways for HCC metastasis, and to profile the unique regulations for HCCs with different etiologies such as HBV and HCV.

## RESULTS

### HCC metastasis-related genes and KEGG pathway analysis

According to text mining and frequency analysis, 1116 genes were identified within 8218 abstracts. The top 20 genes and their frequencies were listed in Table 1. Among these genes, *VEGFA, AFP, CDH1, MMP2* and *MMP9* were mentioned more than 150 times, while *MAPK1, TGFB1, AKT1, CTNNB1* and other genes were also widely studied.

**Table 1:** The top 20 HCC metastasis-related genes based on text mining

Gene	Description	Count
***VEGFA***	vascular endothelial growth factor A	252
***AFP***	alpha fetoprotein	190
***CDH1***	cadherin 1 (E-cadherin)	154
***MMP2***	matrix metallopeptidase 2	154
***MMP9***	matrix metallopeptidase 9	153
***MAPK1***	mitogen-activated protein kinase 1	123
***TGFB1***	transforming growth factor beta 1	118
***AKT1***	AKT serine/threonine kinase 1	110
***CTNNB1***	catenin beta 1	100
***PTK2***	protein tyrosine kinase 2 (FAK)	93
***SPP1***	secreted phosphoprotein 1	85
***NME1***	NME/NM23 nucleoside diphosphate kinase 1	82
***NFKB1***	nuclear factor kappa B subunit 1	76
***MET***	MET proto-oncogene, receptor tyrosine kinase	75
***BSG***	basigin (CD147)	72
***PIK3CA***	phosphatidylinositol-4,5-bisphosphate 3-kinase catalytic subunit alpha	71
***HIF1A***	hypoxia inducible factor 1 alpha subunit	68
***CD44***	CD44 molecule	67
***FN1***	fibronectin 1	65
***HGF***	hepatocyte growth factor	65

Only the genes that co-appeared with metastasis-related phenotypes in the same sentence will be counted. If a gene appeared several times in one sentence, it would be treated once.

KEGG pathway analysis was carried out with identified genes. The top 20 pathways were listed in Table 2, including focal adhesion, adherens junction, regulation of actin cytoskeleton, cytokine-cytokine receptor interaction and so on.

**Table 2:** The most significant KEGG pathways related to HCC metastasis

KEGG Pathway	Genes	*P*-Value
Focal adhesion	98	< 0.0001
Adherens junction	42	< 0.0001
Regulation of actin cytoskeleton	71	< 0.0001
Cytokine-cytokine receptor interaction	78	< 0.0001
MAPK signaling pathway	74	< 0.0001
Toll-like receptor signaling pathway	39	< 0.0001
Oxidative phosphorylation	1	< 0.0001
Apoptosis	32	< 0.0001
Cell cycle	31	< 0.0001
Purine metabolism	6	< 0.0001
Insulin signaling pathway	40	0.0001
Wnt signaling pathway	40	0.0002
Neuroactive ligand-receptor interaction	22	0.0002
TGF-beta signaling pathway	27	0.0002
Pyrimidine metabolism	3	0.002
Tight junction	30	0.002
Glycerophospholipid metabolism	2	0.003
Jak-STAT signaling pathway	37	0.007
Gap junction	24	0.01
Fatty acid metabolism	2	0.01

Official symbols of all 1116 genes were treated as inputs. KEGG pathways were sorted by *P* value.

### HCC metastasis-related PPI analysis

**Figure 1:**
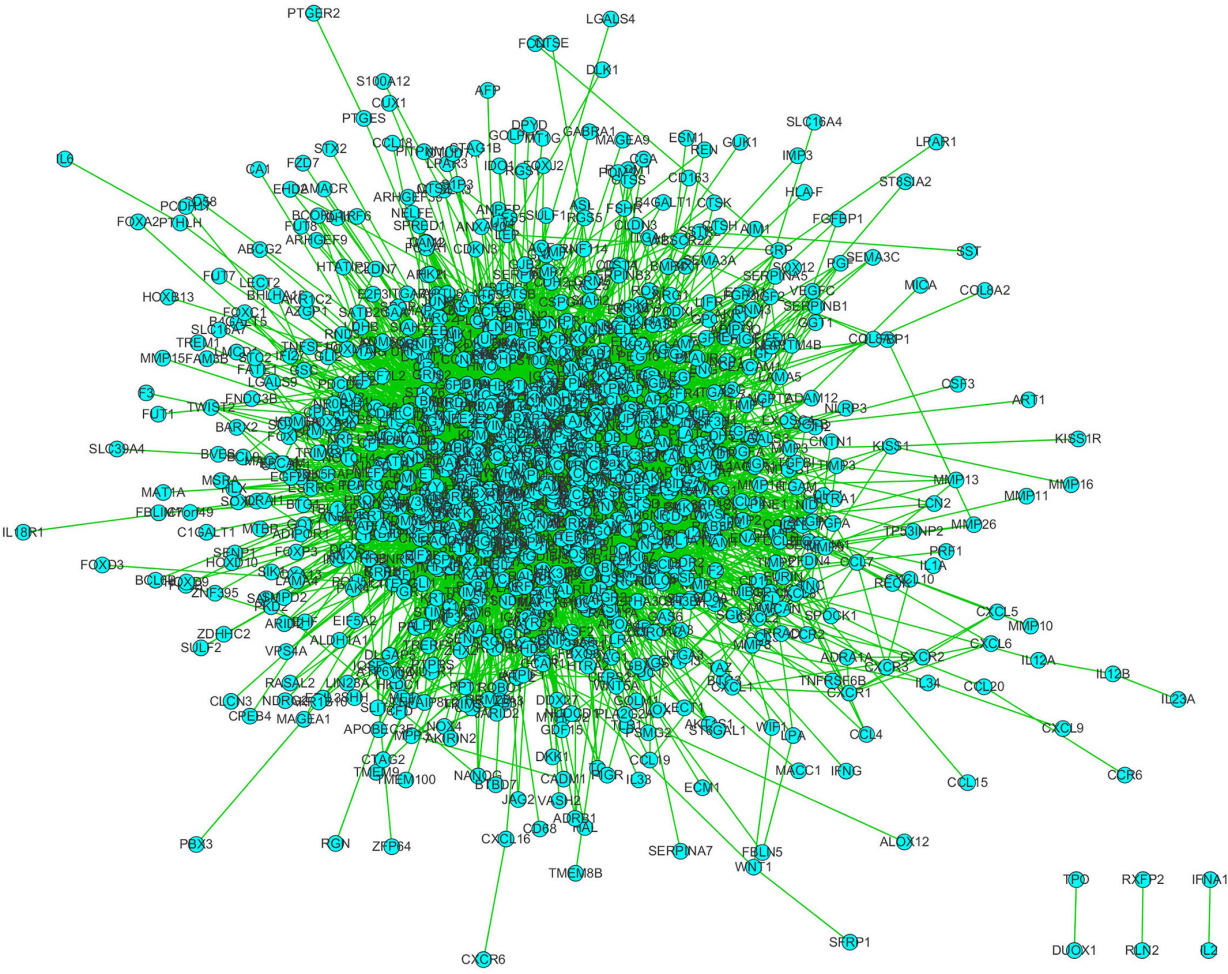
The PPI network of HCC-metastasis related genes All edges were treated as undirected and all interactions were based on experiments. Isolated nodes and self-loops were deleted. Network was built with input nodes only, excluding their neighbours.

Critical regulators usually work as hub proteins in regulation network, so the PPI network among these proteins was generated and illustrated in Figure 1. The degree of nodes (the number of proteins interacting with it) was demonstrated in Table 3. The degree of UBC (ubiquitin C) is much higher than others’, which may partly be attributed to its function in protein degradation. EGFR (degree = 157), MDM2 (degree = 153), TP53 (degree = 152), APP (degree = 149), HSP90AA1 (degree = 149) and other proteins are also predicted as core nodes among the network.

**Table 3:** The top 20 nodes in HCC metastasis-related PPI network

Node	Description	Degree
**UBC**	ubiquitin C	739
**EGFR**	epidermal growth factor receptor	157
**MDM2**	MDM2 proto-oncogene	153
**TP53**	tumor protein p53	152
**APP**	amyloid beta precursor protein	149
**HSP90AA1**	heat shock protein 90 alpha family class A member 1	149
**EP300**	E1A binding protein p300	135
**SUMO1**	small ubiquitin-like modifier 1	130
**GRB2**	growth factor receptor bound protein 2	128
**SRC**	SRC proto-oncogene, non-receptor tyrosine kinase	126
**CTNNB1**	catenin beta 1	125
**FN1**	fibronectin 1	123
**YWHAZ**	tyrosine 3-monooxygenase/tryptophan 5-monooxygenase activation protein zeta	122
**ESR1**	estrogen receptor 1	110
**HSP90AB1**	heat shock protein 90 alpha family class B member 1	107
**HDAC1**	histone deacetylase 1	100
**AKT1**	AKT serine/threonine kinase 1	96
**AR**	androgen receptor	92
**MAPK1**	mitogen-activated protein kinase 1	88
**CUL7**	cullin 7	87
**MYC^†^**	v-myc avian myelocytomatosis viral oncogene homolog	87

The degree of each node was calculated with CytoNCA. All edges were treated as undirected. ^†^MYC was equal with CUL7.

### Metastasis of HCCs resulting from HBV or HCV

Chronic HBV or HCV infections now have been recognized as the major risk factors for the development HCC [4], but they take very different strategies for tumorigenesis. HBV DNA is integrated into the host genome and increases the HCC risk through several approaches such as increased levels of HBV proteins, transactivation of transcription factors, disruption of chromosomal stability and so on [5]. Instead, HCV has no integration into host DNA, neither direct oncogenic activity of its genes. Subsequent HCC always develops following liver fibrosis and cirrhosis [4, 6]. So HCCs caused by HBV or HCV may differ in metastasis mechanisms.

**Figure 2:**
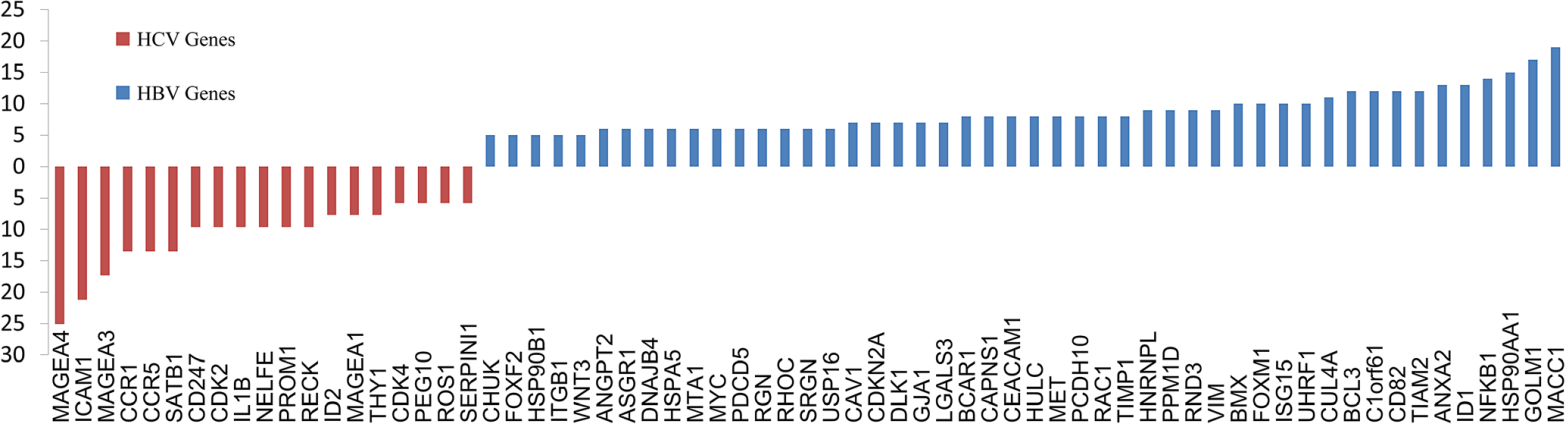
Genes unique to HBV or HCV-related-metastasis Genes with low frequency (freq < 5) were excluded. As papers about HCV-metastasis were less than that of HBV (136 VS 262), all frequencies of HCV particular genes were normalized based on the number of papers (×1.926).

To verify this hypothesis, 286 genes were identified in Pubmed retrieves, 78 of which were shared by HBV and HCV papers, 165 particularly for HBV and 43 particularly for HCV. Genes uniquely mentioned with HBV or HCV were demonstrated in Figure 2, and their KEGG results were listed in Table 4. Besides the overlapping processes, HBV-particular genes were involved in MAPK pathway, tight junction and adherens junction. HCV-particular genes, by contrast, participated in more extensive signaling cascades, including TGF-beta, Jak-STAT, cell cycle, ECM-receptor interaction and gap junction, though the number of them was much less (43 *VS* 165).

**Table 4:** The KEGG pathways for HBV/HCV particular genes related to metastasis

KEGG Pathway	Genes	*P*-Value	For HBV Particular Genes	For HCV Particular Genes
MAPK signaling pathway	15	0.0003	Y	
Tight junction	8	0.004	Y	
Adherens junction	6	0.007	Y	
Cytokine-cytokine receptor interaction	7	0.0003		Y
TGF-beta signaling pathway	4	0.0006		Y
ECM-receptor interaction	3	0.005		Y
Jak-STAT signaling pathway	4	0.005		Y
Gap junction	3	0.007		Y
Cell cycle	3	0.008		Y
Focal adhesion	16/5	< 0.0001/0.005	Y	Y
Regulation of actin cytoskeleton	14/5	0.0003/0.003	Y	Y
Toll-like receptor signaling pathway	7/3	0.006/0.008	Y	Y

As shown in the last two columns, pathways may belong to HBV/HCV-HCC metastasis particular genes, or shared by both.

### Co-occurrence analysis with metastasis-related phenotypes

According to classical theory [7], metastasis can be roughly divided into several steps. Cancer cells have to depart from the original position, invade the extracellular matrix and enter vascular system, where they travel to other sites of body and eventually form new colonization [8, 9]. That is a real tough experience. Reasonably, if a gene can simultaneously affect more than one metastasis related phenotypes, its mutation or de-regulation is more likely to facilitate metastasis.

**Figure 3:**
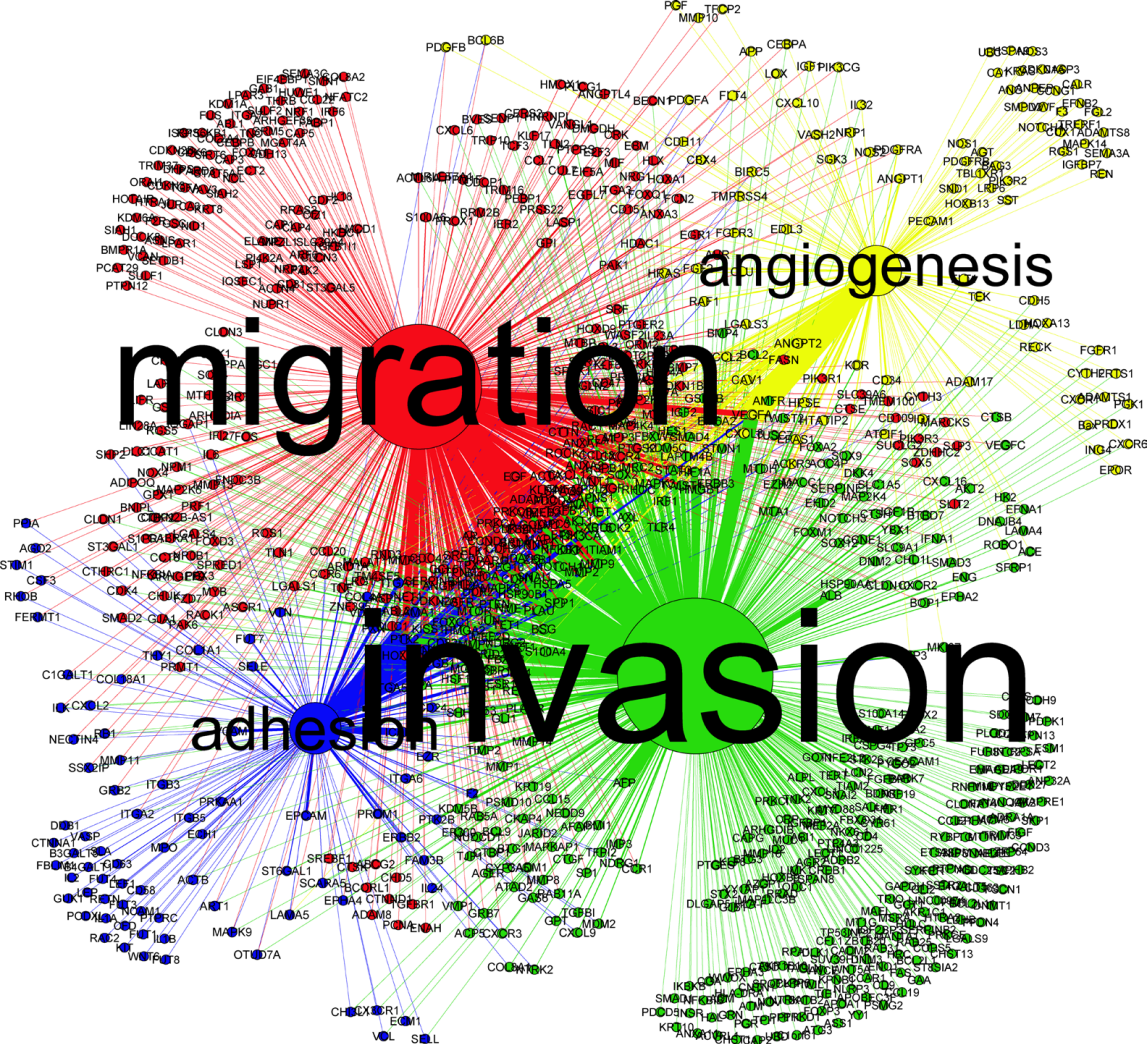
The co-occurrence between genes and metastasis-related phenotypes For each phenotype the size of circle indicated the number of genes that arise with it in one sentence. The thickness of edge reflected the frequency of each co-occurrence relationship.

To identify these genes, four metastasis-related phenotypes were extracted from metastasis cascades. They were *“adhesion”, “migration”, “invasion”* and *“angiogenesis”*. The co-occurrence between these words and genes were examined, and the results were demonstrated in Figure 3. The mostly concerned phenotype was *“invasion”* (freq = 2786), and then *“migration”* (freq = 1826). *“Adhesion”* (freq = 696) and *“angiogenesis”* (freq = 561) had relatively low co-occurrence with genes. Go along with the four typical phenotypes, *PTK2* (with *“adhesion”*, freq = 64), *CDH1* (with *“migration”*, freq = 49), *MMP9* (with *“invasion”*, freq = 104) and *VEGFA* (with *“angiogenesis”*, freq = 98) were the most popular genes, respectively.

**Figure 4:**
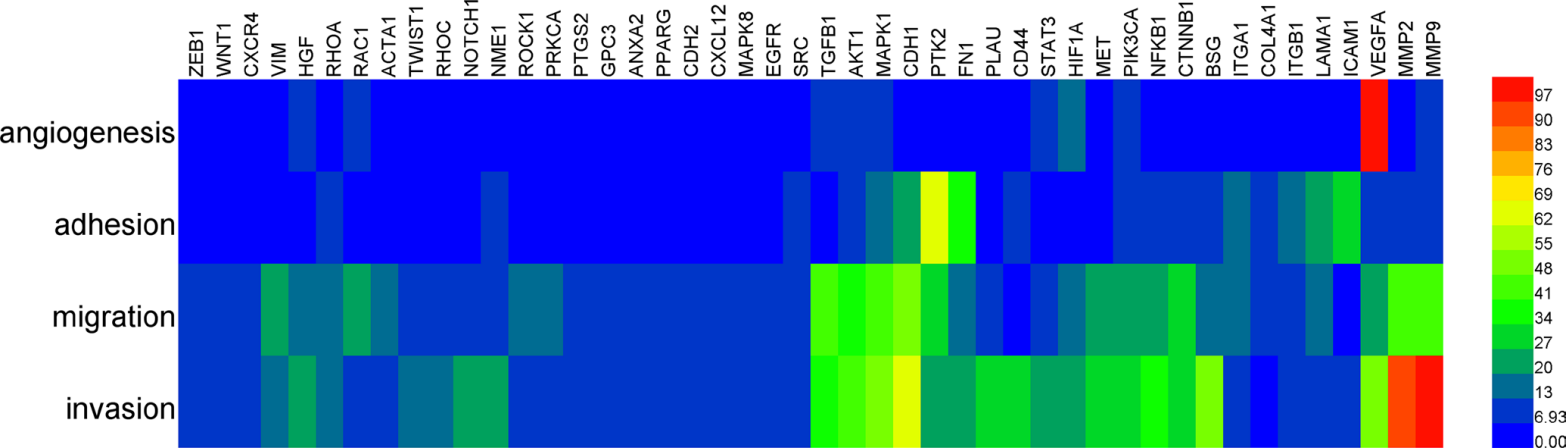
Cluster analysis for genes that co-appeared with metastasis-related phenotypes Data were linearly normalized. Hierarchical cluster analysis was performed based on maximum-linkage, using similarity metric of Euclidean distance.

To highlight the genes that may affect multiple phenotypes, genes co-occurred with 2 or more phenotypes (threshold of frequency was 7) were listed and cluster analysis was performed in Figure 4. Several genes, such as *VEGFA, PI3KCA, MAPK1* and *MMP9*, were obviously involved in almost all steps, and they should be preferentially regarded as potential regulators for HCC metastasis.

## DISCUSSION

As an effective method [10], text mining has been used to explore regulation mechanism for several types of cancers, including glioblastoma [11], endometrial cancer [12], prostate cancer [13, 14], and breast cancer [15]. Actually, the relationship between gene and cancer is specific: under specific environment, through specific pathways, and affect specific processes or phenotypes of tumor biology, such as transformation, proliferation, metastasis, recurrence, drug resistance and so on. To get more valuable information, the objective of text mining should be clearly defined. For our study, we focus on “HCC metastasis”, and the relationship between genes and different steps of metastasis were calculated respectively. With specific aim and multiple methods, the association between genes and HCC metastasis could be more precisely addressed.

The etiology of HCC is another crucial issue. As mentioned above, HBV and HCV infection have different mechanisms of tumorigenesis, so they may exert different approaches for HCC metastasis. Exploration for such etiology-specific pathways may shed promise for targeted drugs and personalized treatments.

As a matter of fact, the most popular genes are not always the most important ones. Meanwhile, some other genes though crucial for HCC metastasis, may not get due attention. The purpose of our paper is not just to gather what have been done by now, but also to reveal what should be focused in the future. Genes with moderate frequency, but involved in multi-processes or interacting with principal molecules, might be fertile land for novel discoveries.

Each method used in this research has its own advantages and disadvantages. Frequency calculation is an effective method for text mining, but not enough for functional analysis. PPI network analysis is useful to find out hub regulators, but the specificity may be impaired with too much inputs. Co-occurrence analysis with phenotypes or biological functions is an improvement of word frequency analysis, where the universality of information and the specificity of the results are simultaneously emphasized, but the feature words have to be identified in advance. So these methods should be carefully selected and results should be extensively considered.

There are still some limitations in this study. First, ABNER is a biomedical entity recognition software based on statistical machine learning [16]. Although it has been optimized, not all genes will be identified. Second, it takes a long time to check gene names, symbols and alias. Third, text mining can calculate the frequencies of specific words and calculate their relationships, but they cannot actually “understand” literatures. However, text mining is still helpful for us to observe molecular biology achievements of HCC metastasis on a macro level, and quantitatively assess the roles and relationships for genes with multidimensional perspectives.

## MATERIALS AND METHODS

We search PubMed with the statement *(HCC OR “hepatocellular carcinoma”) AND metastasis*, and 8218 literatures were found out (to June 15th, 2016). Similarly, “*HBV AND (HCC OR “hepatocellular carcinoma”) AND metastasis”* and “*HCV AND (HCC OR “hepatocellular carcinoma”) AND metastasis*” were applied to collect genes involved in HCC metastasis resulting from HBV or HCV. All abstracts were downloaded from PubMed document retrieval system. Genes and proteins among abstracts were identified with ABNER (Version 1.5) [16, 17]. Gene symbols were normalized manually based on the Entrez Gene Database. Word frequency analysis was performed with Microsoft Excel 2010. To reflect the relationship between genes and metastasis, several phenotypes were selected, such as *“metastasis”, “adhesion”, “migration”, “invasion”* and *“angiogenesis”*. Only the genes that co-appeared with these words in the same sentence will be counted. If a gene appeared several times in one sentence, it would be treated once. KEGG pathway analysis was performed on GATHER (Gene Annotation Tool to Help Explain Relationships, http://gather.genome.duke.edu/) [18], and the threshold of *P* is 0.05.

The protein-protein interaction (PPI) network was integrated with BisoGenet [19], and interaction data come from BIND [20, 21], BioGrid [22], DIP [23], MINT [24], IntAct [25] and HPRD [26]. All interactions were based on experiments. The PPI network was illustrated with Cytoscape (version 3.4.0) [27] and analyzed with CytoNCA [28], a plugin for Cytoscape. Co-occurrence analysis between genes and phenotypes was illustrated with Gephi (Version 0.8.2 beta) [29]. Hierarchical cluster analysis based on maximum-linkage (similarity metric with Euclidean distance) was performed with HemI (Version 1.0) [30].
